# Grando or Chalazion in an Eagle

**Published:** 1882-10

**Authors:** Wm. A. Conklin


### V.—GRANDO OR CHALAZION IN AN EAGLE.

REPORTED FROM THE CENTRAL PARK ZOOLOGICAL GARDEN, BY WM. A CONKLIN, D.V.S.

The history of this case is unfortunately quite brief. The principal thing was the development of a large tumor and a small secondary tumor over the eye, which partially closed the opening of the eyelid. It also crowded the eye downward and outward.

The larger of the two tumors was about 2.5 centimetres or (one inch) in diameter, the smaller about one-third that size.

Upon microscopical examination the growth was found to have an incomplete capsule, within which there was a series of concentric laminæ. These laminæ, when examined with a high-power lens, were found to be composed of a few filaments of incompletely formed fibrillated elements, but mostly of a granular debris.

[This is the first case of the kind that we have met with, but Dr Conklin informs us that this trouble is quite common among eagles, and has caused considerable trouble in the Philadelphia Zoological Garden.

If our diagnosis is substantiated by more cases, it seems reasonable to suppose that an early operation would ca.se a disappearance of the tumor and a permanent cure.

The growths are undoubtedly in or near the under surface of the tiarsal cartliges primarily. By everting the eyelid, making an incision into the sac of the tumor and turning out its contents, it seems reasonable to suppose that when practiced early in their development, a permanent cure might be effected.—ED].

